# Pre-weaning dietary iron deficiency impairs spatial learning and memory in the cognitive holeboard task in piglets

**DOI:** 10.3389/fnbeh.2015.00291

**Published:** 2015-10-30

**Authors:** Alexandra Antonides, Anne C. Schoonderwoerd, Gabi Scholz, Brian M. Berg, Rebecca E. Nordquist, Franz Josef van der Staay

**Affiliations:** ^1^Emotion and Cognition Group, Department of Farm Animal Health, Faculty of Veterinary Medicine, Utrecht UniversityUtrecht, Netherlands; ^2^Brain Center Rudolf Magnus, Utrecht UniversityUtrecht, Netherlands; ^3^Faculty of Technique and Life Sciences, Institute of Applied Sciences, Professional University HAN (Hogeschool Arnhem & Nijmegen)Arnhem, Netherlands; ^4^Department of Global Discovery, Mead Johnson Pediatric Nutrition InstituteEvansville, IN, USA; ^5^Division of Nutritional Sciences, University of Illinois at Urbana-ChampaignUrbana, IL, USA

**Keywords:** iron deficiency, cognition, development, growth, spatial learning, memory, hematology, pigs

## Abstract

Iron deficiency is the most common nutritional deficiency in humans, affecting more than two billion people worldwide. Early-life iron deficiency can lead to irreversible deficits in learning and memory. The pig represents a promising model animal for studying such deficits, because of its similarities to humans during early development. We investigated the effects of pre-weaning dietary iron deficiency in piglets on growth, blood parameters, cognitive performance, and brain histology later in life. Four to six days after birth, 10 male sibling pairs of piglets were taken from 10 different sows. One piglet of each pair was given a 200 mg iron dextran injection and fed a control milk diet for 28 days (88 mg Fe/kg), whereas the other sibling was given a saline injection and fed an iron deficient (ID) milk diet (21 mg Fe/kg). Due to severely retarded growth of two of the ID piglets, only eight ID piglets were tested behaviorally. After dietary treatment, all piglets were fed a balanced commercial pig diet (190–240 mg Fe/kg). Starting at 7.5 weeks of age, piglets were tested in a spatial cognitive holeboard task. In this task, 4 of 16 holes contain a hidden food reward, allowing measurement of working (short-term) memory and reference (long-term) memory (RM) simultaneously. All piglets received 40–60 acquisition trials, followed by a 16-trial reversal phase. ID piglets showed permanently retarded growth and a strong decrease in blood iron parameters during dietary treatment. After treatment, ID piglets' blood iron values restored to normal levels. In the holeboard task, ID piglets showed impaired RM learning during acquisition and reversal. Iron staining at necropsy at 12 weeks of age showed that ID piglets had fewer iron-containing cells in hippocampal regions CA1 and dentate gyrus (DG). The number of iron-containing cells in CA3 correlated positively with the average RM score during acquisition across all animals. Our results support the hypothesis that early-life iron deficiency leads to lasting cognitive deficits. The piglet as a model animal, tested in the holeboard, can be useful in future research for assessing long-term cognitive effects of early-life diets or diet-induced deficiencies.

## Introduction

The effects of nutrition on development and health has received much attention in the past decades. Good nutrition is essential for development and growth, physical health, mental health, and well-being. Malnutrition can be defined as any disorder of nutrition, such as over nutrition, under nutrition and unbalanced nutrition, with one or more micronutrient or mineral deficiencies (Allison, 2000). Nearly a third of the world population is suffering from one or more forms of malnutrition, and around half of the deaths among children under the age of five are associated with it (de Onís et al., 1993). Consequences of malnutrition include impaired mental and physical development, disability, and death. The most common nutritional deficiency is iron deficiency, affecting more than two billion people of all ages worldwide (WHO, 2000; Ramakrishnan and Yip, 2002). Iron is an essential micronutrient and is required for many biological functions in the body, such as gene regulation, binding oxygen and serving as a cofactor in many enzymes. Furthermore, iron plays an important role in neural functioning, for example in myelination, neurotransmission and energy metabolism (Youdim et al., 2010). The severity of and specific impairments caused by iron deficiency are dependent on the timing of the deficiency. There are three periods in human development with an elevated risk for iron deficiency: the early neonatal period, toddlerhood, and adolescence in females (Georgieff, 2011). The most common period for iron deficiency in humans is between zero and 5 years of age (McLean et al., 2009), thus during early development. Studies have shown that children who suffered from early iron deficiency (from the late fetal period through 2 years of age) perform worse in motor, cognitive, social and emotional tasks compared to controls that were fed a balanced diet (Walter et al., 1989; Lozoff and Georgieff, 2006). In a longitudinal follow-up study, children who had suffered from severe iron deficiency in infancy still showed behavioral and developmental deficits—such as reduced mental and motor functioning, social and attentional problems—10 years after iron treatment (Lozoff et al., 2000).

In order to elucidate the biochemical and neurophysiological mechanisms underlying iron deficiency, the use of rodent iron deficiency models has increased in the recent past. Such studies have shown that iron deficiency causes numerous abnormalities on the morphological, neurochemical and behavioral level. Deficits resulting from iron deficiency suggest impairments specifically in the prefrontal cortex (PFC) and hippocampus (Rao et al., 2013). The hippocampus is known to be involved in spatial learning and memory (Bird and Burgess, 2008). Indeed, hippocampal dysfunctions impair spatial learning and memory (Olton et al., 1978; Lopes da Silva et al., 1986). Compromised spatial orientation learning has also been found in cognitive and behavioral iron deficiency studies. For example, a study on the effects of maternal iron deficiency on hippocampal development in mice pups found impaired spatial memory function, reduced neurogenesis and reduced volumes of the hippocampus in iron deficient (ID) animals (Ranade et al., 2013). In the Morris water maze test, a spatial learning and memory task, ID rats took longer to find the hidden platform compared to controls (Yehuda et al., 1986). Similar results were found for rats that suffered from perinatal iron deficiency when tested in adulthood after iron repletion, showing that iron repletion after early iron deficiency does not reverse iron deficiency induced impairments (Bourque et al., 2008). Perinatal iron deficiency in rats causes differential regional losses of brain iron content, with the most profound declines found in the hippocampus and prefrontal cortex (Siddappa et al., 2003). Iron deficiency extended to the end of weaning in rat pups caused brain iron concentration to decline and delayed the learning of sensorimotor skills (Beard et al., 2006). Interestingly, most of these abnormalities do not recover after iron repletion (Yehuda and Youdim, 1989; Bourque et al., 2008; Ranade et al., 2013). This suggests that iron deficiency during early development causes irreversible impairments in brain structure and function. These impairments are believed to be due to rapid brain growth during early development and its associated high iron demand (Lozoff and Georgieff, 2006).

Mice and rats are the most commonly used animal model species in neurodevelopmental research (Clancy et al., 2007). However, these rodents have a different timing of early development than humans, both pre- and post-natally. For example, the developing brain of rats is believed to be comparable to that of a newborn human baby, with regard to the degree of maturation, around postnatal day 12–13 (Romijn et al., 1991). Because of developmental differences and differences in brain morphology between rodents and humans, more and better suited animal models are needed to study the long-term cognitive effects of iron deficiency in a controlled manner. Other animal models for studying cognitive effects of iron deficiency have been suggested that more resemble the early development of humans, including guinea-pigs (Fiset et al., 2015), pigs (Rytych et al., 2012), and primates (Golub et al., 2007).

In comparison to other animals mostly used in neurotranslational research, the brain growth spurt and pattern of brain development in pigs most resembles that of humans (Dobbing and Sands, 1979; Conrad et al., 2012). The pig has a relatively large brain that is, like the human brain, gyrencephalic (with cerebral convolutions), whereas the rodent brain is lissencephalic (smooth surface). Although a difference in size of left and right hippocampi is found in humans but not in pigs, MRI studies have shown that hippocampi of both species show a similar sex-dependent growth trajectory (Conrad et al., 2012). Additionally, the distribution of gray and white matter is comparable between pigs and humans (Lind et al., 2007). Pigs are social and intelligent animals which can be trained in complex cognitive tasks (Mendl et al., 2010). Moreover, the pig is a precocial species, and thus can be weaned almost immediately after birth. This has the advantage that behavioral tasks can be conducted with piglets at as young as 1 or 2 weeks of age (Dilger and Johnson, 2010). Furthermore, pigs are easily available with well-documented life history information available.

Pigs can thus serve as a promising model animal to examine environmental effects during the neonatal period, when the brain is rapidly developing, on learning and memory later in life.

Furthermore, the resemblance of the pig's digestive system and metabolic processes to those of humans makes the pig suitable model species for studying effects of nutrition (Puiman and Stoll, 2008). In particular, pigs may be useful to study the effects of iron deficiency, as they have limited iron stores at birth, a high requirement of iron and a low external supply of iron (Starzyński et al., 2013). The ancestor of the domesticated pig, the wild boar, copes with its high iron needs by rooting and thus ingesting iron from soil. Piglets reared in confinement do not have access to this natural source of iron. Moreover, sow's colostrum and milk do not meet the piglets' high iron requirements, even if the sow is fed a high iron lactation diet or administered an injection of iron dextran (Brady et al., 1978). In commercial pig farming, it is therefore common practice to provide piglets with an intramuscular injection of iron dextran on day 3–6 after birth (Svoboda and Drabek, 2005).

Studies investigating iron deficiency in pigs have mainly looked at the underlying molecular mechanisms or investigated the prevention and correction of iron deficiency (Lipiński et al., 2013; Starzyński et al., 2013). A recent study that looked at the effects of dietary induced iron deficiency on learning and memory in neonatal piglets, found that severe ID piglets could not acquire a food motivated double T-maze task, whereas mild ID piglets showed deficits in reversal learning (Rytych et al., 2012). However, the performance of ID pigs was assessed during the period of dietary ID treatment, and not during or after iron repletion. The present study investigated the effects of severe pre-weaning iron deficiency on cognitive performance after iron deficiency treatment in piglets later in life. To this end, we used the spatial cognitive holeboard task for piglets (Arts et al., 2009; Gieling et al., 2012). In this task, four hidden rewards can be found in a matrix of 4 × 4 possibly rewarded sites, allowing for working memory (WM) and reference memory (RM) to be measured simultaneously. WM is a form of short-term memory that is used during a specific trial (van der Staay et al., 2012) and that, once used, should be forgotten (Dudchenko, 2004). This includes, for example, which sites an animal has already visited during a trial, i.e., this information is relevant only within a trial. RM is a form of long-term memory that involves the general rules of a task, such as where to find hidden rewards (van der Staay et al., 2012). Information stored in RM is relevant across trials. Additionally, effects of pre-weaning iron deficiency on blood iron parameters during and after treatment, and brain histology after iron repletion was investigated.

We expected that ID piglets would show a decline in blood iron values during dietary iron deficiency treatment, and impaired performance in the holeboard task compared to the control group, by making more errors in the task (i.e., showing lower memory scores) and taking more time to complete the task. Furthermore, as brain iron concentration has been shown to be normalized in rodents after 2 weeks of iron repletion (Erikson et al., 1997; Piñero et al., 2000), we expected to find no effect of treatment on the pigs' brain iron concentration at 12 weeks of age.

## Materials and methods

### Ethics note

This study was reviewed and approved by the local ethics committee (DEC, DierExperimenten Commissie) and was conducted in accordance with the recommendations of the EU directive 86/609/EEC. All efforts were made to minimize the number of animals used and to avoid suffering.

### Subjects

Ten sibling pairs of male piglets were selected from 10 different litters [(Terra × Finnish landrace) × Duroc] at the commercial pig breeding farm of Utrecht University, Netherlands. This was done in two runs with 2 weeks in between to ensure that enough male piglets with similar average weights could be selected. During the first days, all piglets were allowed to ingest colostrum from the sow in order to trigger their immunocompetence (Rothkötter et al., 2002). This was believed to have little to no influence on the iron status of the piglets, because of the low iron content in sow colostrum and milk (Brady et al., 1978). During this time, no creep feed was provided. From each litter, two healthy male piglets with birth weights closest to the average birth weight of the entire litter were selected (51.3 ± 3.9% of the piglets within the 10 litters was male). One piglet of each pair was randomly assigned to the ID treatment group, the other to the control group. Four to six days after birth, selected piglets were separated from the sow and transported to the experimental facilities, which was on the same day for all piglets per run in order to reduce mixing stress. In the first week after arrival, the animals were weighed daily to closely monitor weight gain, after which they were weighed weekly. Two ID piglets were euthanized during the first week after arrival, as they did not consume sufficient amounts of milk formula and therefore did not gain weight. Thus, the experiment was conducted with 8 ID piglets and 10 control piglets. The experiment was performed blind, in such a way that neither the experimenters nor the animal caretakers or other people directly involved in this research were aware which piglets underwent the ID treatment, and which piglets served as controls. The treatment groups were unblinded after the statistical analyses were completed.

### Housing

The piglets were housed in a multifunctional room in the clinic of the Department of Farm Animal Health (Utrecht University, Netherlands) in four adjacent identical pens, each measuring 1.25 × 2.50 m. They were housed in groups of five pigs (one ID group consisted of three animals after two animals were euthanized due to lack of growth, which was 1 week after arrival), sorted by treatment group and age. The concrete floor of the pens was covered with a layer of sawdust and straw. Chains, rope and plastic balls were offered as enrichment and were changed regularly. When the animals were ~10 weeks old, the pens became too small for the piglets according to the guidelines for housing pigs of that age (Forbes and Associations, 2007). It was thus decided to rehouse the piglets in pens measuring 2.50 × 2.50 m, which required moving the animals to an adjacent, identical room. Rehousing was done in two runs, so that it was performed at the same age in both groups. As the animals were led to the experimental room and back daily for testing, this was expected to have no effect on behavioral performance in the holeboard task. Room temperature was gradually decreased from 26°C in the first week to 20°C at the end of the experiment. During the first week, a heating lamp was hung 1 m above the floor in each pen. This was done to ensure the piglets would not chill, as young piglets' thermoregulatory abilities are low (Herpin et al., 2002). The room had a light-dark regime of 12:12 h with lights on at 7 a.m. A radio was playing continuously at a moderate volume to mask environmental noise, slightly louder during daytime (7 a.m. to 4 p.m.) than at night.

### Treatment

The control group received a needle-free injection of 1 ml iron dextran containing 200 mg iron (MS FerroPig, Schippers Export B.V., Netherlands) on day 4–6 after birth (directly before transport to the experimental facilities). The treatment group was administered 1 ml of saline (NaCl) in the same manner. As the experiment was performed blind, this was done via a third person, who was informed about the coding of the treatment groups and experimental diets (blinded to the experimenters) and communicated this with the animal caretakers who applied the injections. After arrival in the experimental facilities, the piglets were fed one of two custom diets prepared by the Mead Johnson Pediatric Nutrition Institute Technical Center (Evansville IN, USA). The control group was fed a balanced milk replacer, formulated to achieve an iron content of 100 mg Fe/kg formula. The treatment group received an ID diet formulated to achieve 10 mg Fe/kg, but balanced for all other nutrients. The actual iron content in samples was determined at 88 mg Fe/kg diet for the balanced formula and 21 mg Fe/kg diet for the ID formula.

**Figure 1:**
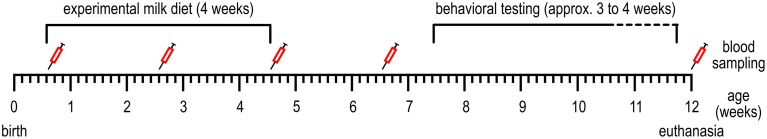
**Timeline of the events conducted during the experiment**. See Table 1 for more detailed information.

Experimenters and animal caretakers were blinded to the iron content of the formulas, which were provided with a code on the packaging by the supplier. Milk replacer was freshly prepared five times per day; at 7.30 a.m., 11 a.m., 2 p.m., 4 p.m., and 9 p.m. and fed in two feeding bowls per pen (from 2 weeks of age in two troughs, to reduce competition). The amount of milk replacer fed was adjusted daily to meet the consumption of the animals and ranged from 1 to 4 L per feeding. The piglets were fed these diets for 28 days (for a timeline, see Figure 1; Table 1). Then, all animals received the same commercial piglet feed *ad libitum* (containing 190–240 mg Fe/kg). The animals were weaned gradually: during the first week of weaning (day 28–35 after the start of the experimental diet) in addition to the commercial piglet feed, the assigned milk replacer was still provided (6 L on the first day of weaning, which was gradually decreased to 2 L at the end of weaning). Water was available *ad libitum*.

**Table 1:** **The timing of events conducted during the experiment, relative to the birth of the piglets (middle column) and relative to weaning and the start of the experimental diets (right column)**.

**Timing of the conducted events during the experiment**		
**Event**	**Conducted at (age in days** ±**1)**	**Timing relative to start of experiment (i.e., to transport to experimental facilities)**
Birth	0 days	−5 (± 1) days
Iron dextran/saline injection	5 days	0 days (0 weeks)
Experimental diet	5 days until 33 days	0–28 days (0–4 weeks)
Gradual transition to regular feed	33 days until 40 days	28–35 days (4–5 weeks)
Blood sampling	5, 19, 33, 47, 85 days	0 days (0 weeks), 14 days (2 weeks), 28 days (4 weeks), 42 days (6 weeks), and 80 days (~11.5 weeks)
Start of behavioral testing	54 days	49 days (7 weeks)
End of behavioral testing	Between 77 days (min. 40 acq. trials) and 83 days (max. 60 acq. trials)	Between 72 days (10 weeks; min. 40 acq. trials) and 78 days (11 weeks; max. 60 acq. trials)
Euthanasia and brain histology	85 days	80 days (~11.5 weeks)

### Blood sampling

On the day of transport to the experimental facilities (age 4–6 days, see Table 1), blood was collected from the ear veins of the piglets to determine blood hematocrit and hemoglobin levels, using the The epoc® Reader and Host Mobile Computer (Alere Inc., Waltham MA, USA). At 2, 4, and 6 weeks after the start of the experimental diet and at 12 weeks of age (Figure 1), due to difficulties with collecting blood from the ear veins, blood samples were taken from the jugular artery (3.5 ml) to determine hematocrit, hemoglobin and serum iron values. Blood hemoglobin and hematocrit were determined using the Siemens ADVIA® 2120i System with ADVIA Multispecies Testing software. Serum iron was determined using the Beckman Coulter UniCel DxC 600 according to standard procedures.

### Apparatus

**Figure 2:**
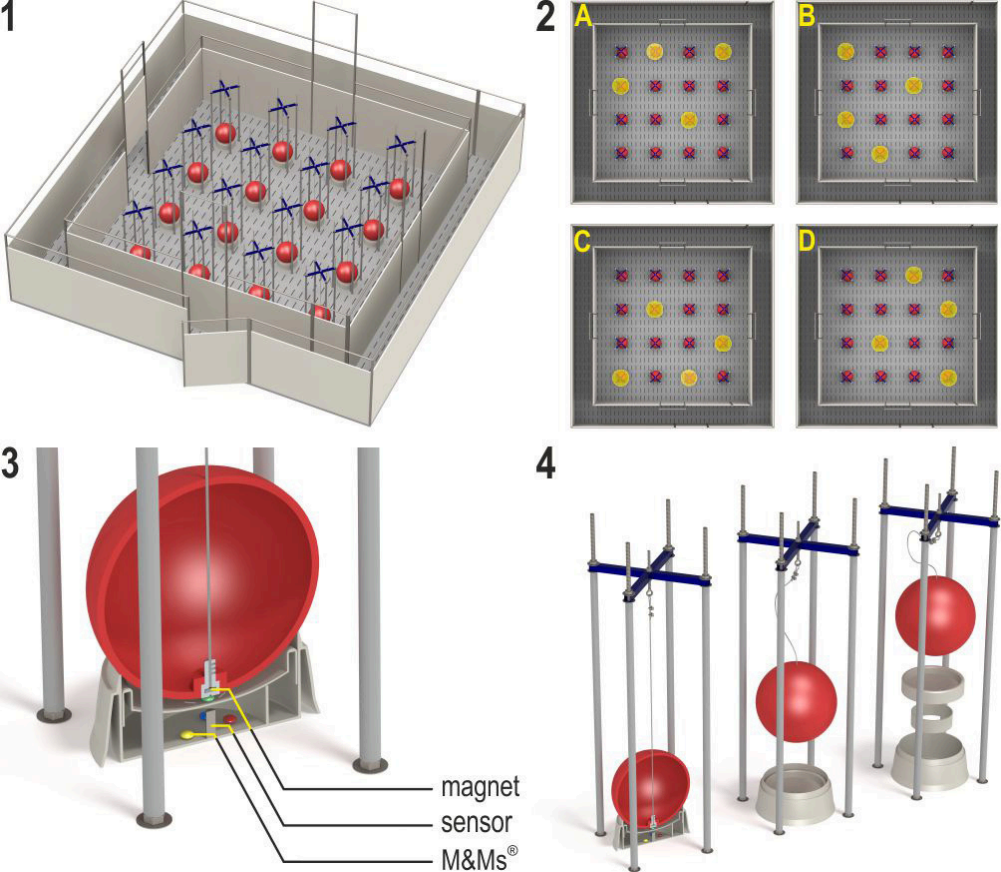
**(1)** The spatial cognitive holeboard for pigs. **(2)** The four patterns of baited holes. **(3)**, **(4)** Constructional details of the holes. Each hole—a fool bowl with a false bottom under which three M&M's® chocolates are placed in order to mask odor cues—is covered by a red ball. Each food bowl is equipped with a sensor that sends a signal to the computer if the contact between the magnet in the ball is interrupted; i.e., when the pig lifts the ball with its snout (illustrations: Yorrit van der Staay).

The holeboard apparatus (Ossendrijver BV, Achterveld, Netherlands) consisted of a square arena of 360 × 360 cm with a 4 × 4 matrix of food bowls, surrounded by a small corridor (40 cm) with a slatted black synthetic floor. The synthetic walls were 80 cm high and had a steel bar on top (total height: 1 m). The apparatus was elevated 25 cm off the floor. The arena could be entered through four different guillotine doors, one on each side. The entry door was randomly assigned in each trial and was operated from outside the arena using a rope and pulley system. Pigs entered the holeboard through the main entrance and always turned left into the corridor until they found an open door, through which they entered the testing arena (Figure 2, panel 1). Piglets inside the holeboard arena were able to see the surrounding walls of the experimental room and the ceiling with two rows of fluorescent tubes, as well as the two experimenters standing in front of the holeboard to the right of the main entrance door. The experimenters avoided eye contact with the piglets during trials. Auditory extra-maze cues were a radio that was playing continuously during testing hours, and the piglet's pen mates in the waiting area in front of the holeboard apparatus, where they were housed during testing.

The 16 food bowls were covered by red plastic balls which could be lifted by the piglets with their snout (JollyBall Dog Toy, ø 24 cm, 400 g), to prevent the piglets from finding the rewards by sight. To ensure that the rewards were not found by smell, every food bowl contained three rewards (replaced daily) under a false bottom (Figure 2, panel 3). The apparatus was cleaned with a water hose at the end of each testing day and after a trial if an animal had defecated.

Hole visits were automatically recorded using custom made software (Blinq Systems, Delft, Netherlands). A visit was scored when a pig lifted the ball and the connection between the magnet in the ball and the sensor in the food bowl was broken (Figure 2, panel 3). This signal was registered by an interface (LabJack) and sent to a PC. An iron wire attached to the top of the ball ensured that the ball would always fall back into place with the magnet located directly above the sensor (Figure 2, panel 4). A visit was not counted as a revisit when the same ball was lifted within 10 s and no other holes were visited in between. A trial started when a pig entered the arena with both front legs and ended when a piglet found all four rewards or when the maximum time of 450 s was reached, whichever event occurred first.

### Behavioral testing

During the weeks after arrival and before testing started, all piglets were gradually habituated to the experimenters, hallway and holeboard apparatus in two sessions of 30 to 60 min per day. At the start of holeboard habituation, all balls were lifted and in each of the 16 bowls multiple mini-marshmallows were placed as reward, as these were easy to consume for the young animals. When all animals had been seen consuming rewards from the bowls, all balls were lowered in order for the piglets to learn to lift the balls to find rewards. Habituation was done with all piglets of a pen in the arena, then in groups of two or three and eventually individually. During this time, pen mates were present in the waiting area in front of the holeboard, thus piglets in the holeboard could still hear and smell their pen mates. Piglets that seemed to have trouble learning to lift the balls in order to find rewards were allowed longer habituation sessions, in order to ensure that all piglets had roughly the same level of performance in finding rewards before testing in the holeboard started. At the end of the habituation period and during holeboard testing, M&M's® chocolates were used instead of mini-marshmallows. Holeboard testing started when all piglets had learned to search for rewards while being alone in the holeboard, which was when the piglets were ~7.5 weeks old (Figure 1).

All piglets received six individual habituation trials (two per day) in which all holes were baited. Then, the animals received two trials in close succession per day (massed trials) for the first four testing days, after which they received four massed trails per day, in sets of two trials in close succession each. Each piglet was assigned its own configuration of baited holes, in which 4 of the 16 holes were baited (Figure 2, panel 2). In total, four different configurations were used, in such a way that every hole was baited equally often. All piglets received 40–60 acquisition trials (12–17 testing days) and 16 reversal trials (4 testing days). A piglet was switched to the reversal phase when its RM score averaged above 0.7 over the last four trials (1 day) after at least 40 acquisition trials. After a maximum of 60 acquisition trials, all pigs were switched to the reversal configuration, regardless of their performance. The reversal configuration was the 180° rotated pattern of baited holes used during acquisition, i.e., a change from configuration A to C, B to D, C to A, or D to B. All piglets received 16 reversal trials, thus all piglets received a minimum of 56 trials and a maximum of 76 trials during the acquisition and reversal phase.

### Brain histology

Due to the unexpected death of one control animal at 11 weeks of age, brain sections of 9 control animals and 8 ID animals were analyzed. At the end of the experiment (at 12 weeks of age), all animals were euthanized by an intracardial injection with an overdose of pentobarbital (Euthasol®, AST Farma B.V. Oudewater, Netherlands). Directly afterwards, brains were dissected and weighed. Both hippocampi were then carefully removed and weighed, after which the left hippocampus was cut in half and the dorsal part was stored in 4% phosphate buffered formaldehyde, pH 7.0 (Klinipath, Duiven, Netherlands) at 4°C, which was refreshed after 2 h and within 24 h replaced with 70% ethanol. Samples were then stored at 4°C until slicing. Brains were sliced in 0.12 M phosphate buffer saline on ice using a vibratome (VT 1200S Leica Biosystems, Nussloch, Germany). Dorsal hippocampal samples were sliced in such a way that in each container the distance between the sections was 240 μm. Sections were collected in series of six sections and stored in tubes of 0.12 M phosphate buffer saline (PBS) and 0.1% sodium azide at 4°C. Staining was done using the free-floating method; 40 μm sections were stained in baskets with eight compartments. PBS was used as a negative control. All steps were performed using a tilt shaker (WS-10, position 10, Edmund Buehler Gmbh, Hechingen, Germany). Sections were washed in 0.05 M tris-buffered saline. Staining took place in the dark to prevent influence by light on immunohistochemical reactions.

Iron staining was done using a commercial kit (Prussian blue iron stain kit, Polysciences Inc., Warrington, USA). Sections were washed with 0.12 M PBS, incubated in a solution of 4% potassium ferrocyanide and 4% hydrochloric acid (1:1) for 45 min at room temperature, then washed with AD. The reaction was intensified with 3,3′-Diaminobenzidine tetrahydrochloride DAB catalyzed by H_2_O_2_ (Sigma-Aldrich, 1 ml DAB stock + 1.5 ml TBS + 2.0 μl H_2_O_2_) for 5 min. Afterwards, sections were mounted on superfrost slides (Thermo Scientific, Braunschweig, Germany) and dried overnight at 37°C followed by dehydration through graded alcohol series (70 to 100%) and xylene. Slides were then embedded with DePeX (Serva electrophoresis, Heidelberg, Germany) and coverslipped. Four hippocampal areas of interest were chosen: CA3, CA1, DG, and subiculum (sb). The CA3 region is associated with spatial pattern recognition and short-term memory; the CA1 region is involved in short- and intermediate term memory and temporal pattern separation (Kesner et al., 2004). Both CA3 and CA1 are involved in signaling the animal's presence in particular regions of space (i.e., self-location; Barry and Burgess, 2014). The DG is thought to be involved in fine spatial information processing (Kesner et al., 2004; Hunsaker et al., 2008), and the subiculum in memory retrieval and spatial encoding (Stafstrom, 2005).

Visual scoring was performed using a microscope (Olympus Bx40) at 10 × 0.25 magnification. An ocular counter was used where a randomized sequence of 20 squares out of 100 squares (unit of 1 square: 1 mm^2^) was scored. Iron-containing cells were visible as brown dots, which were counted per mm^2^ and averaged for each hippocampal area over five sections per animal. Scoring was done with coded sections, in such a way that the person that scored the sections did not know which animal of which treatment group was scored (blind procedure).

### Statistical analyses

All analyses were performed using the statistical software SAS (version 9.4, SAS Institute, Cary, NC, USA). Normal distribution of all variables was assessed using the Shapiro-Wilk test (SAS PROC UNIVARIATE). All variables expressing latencies or durations were log_10_-transformed to meet the normality assumption.

Birth weights of control and ID animals used in the experiment were compared using a mixed model ANOVA with litter as random effect. The effects of treatment on the growth curves were analyzed with a mixed model ANOVA to account for clustering of piglets within litters and repeated measurements within piglets, with the fixed effects Treatment (ID or control), Week, and the Treatment by Week interaction.

From the holeboard trials, the measures in Table 2 were calculated (van der Staay et al., 2012). These measures were analyzed using the mean of four trials, resulting in trial blocks. The first 40 acquisition trials thus divided into trial blocks 1–10 were analyzed, yet not the extra acquisition trials that a piglet received when it had not yet reached the criterion of RM > 0.7 after 40 trials. The following 16 reversal trials were also analyzed in blocks of four trials, thus divided into four trial blocks (trial blocks 11–14). The holeboard data analyses were performed for three different phases: acquisition, transition and reversal. The transition phase is the switch from the acquisition phase to the reversal phase, i.e., the last trial block of the acquisition compared to the first trial block of the reversal (trial block 10 compared to trial block 11). This is a measure of the response flexibility of an animal: a large difference means that the animal faced difficulties to adapt to the new situation.

**Table 2:** **Measures recorded or calculated in the holeboard task**.

**Holeboard task measures and their definition**	
**Measure**	**Definition**
Working memory (WM)	A ratio defined by the number of visits that yield a food reward divided by the number of visits and re-visits to the rewarded set of holes
Reference memory (RM)	A ratio that is defined by the number of visits and re-visits to the rewarded set of holes divided by the number of visits and re-visits to all holes
Trial duration (TD)	The time between entering the holeboard and finding all four rewards (when not all rewards were found the maximum trial duration of 450 s was recorded)
Inter-visit interval (IVI)	The average time between two hole visits during a trial
Latency to the first visit (LFV)	The latency until the first visit during a trial
Latency to the first reward (LFR)	The latency until the first rewarded visit during a trial
Total visits (TV)	The total number of hole visits made during a trial
Unrewarded visits (URV)	The total number of unrewarded hole visits made during a trial
Rewarded visits (RV)	The total number of rewarded hole visits made during a trial
Number of visits until 1st reward (Vfirst)	The number of hole visits until the first reward was found
Number of visits until 2nd reward (Vsecond)	The number of hole visits between the first and second reward were found
Number of visits until 3rd reward (Vthird)	The number of hole visits between the second and third reward were found
Number of visits until 4th reward (Vfourth)	The number of hole visits between the third and fourth reward were found

*For an explanation of the “number of errors per reward” measures, see Gieling (2013), pp. 173–176. In short, it is the number of errors that were made before the next reward was found*.

Effects of treatment on performance in the habituation trials in the holeboard (six successive trials preceding testing in which all holes were baited), on the learning curves of the acquisition phase (ten successive trial block means of four trials each) and reversal phase (four successive trial block means of four trials each), and on the transition between the acquisition and reversal phase, were analyzed using mixed model ANOVAs. For holeboard habituation trials, fixed effects were Treatment, Trial and the Treatment by Trial interaction. For the holeboard acquisition, transition and reversal phase, fixed effects were Treatment, Trial blocks and the Treatment by Trial blocks interaction.

Treatment effects on blood hematocrit, hemoglobin and serum iron were analyzed for five time points (0, 2, 4, and 6 weeks after the start of treatment and at 12 weeks of age, see Figure 1) using mixed model ANOVAs. Fixed effects were Treatment, Week and the Treatment by Week interaction (Note that the number of observations differed per blood collection moment and per variable due to technical difficulties during either the blood collection or the analyses; for the number of observations per time point and per variable see Supplementary Table 1). In case of significant interaction effects of Treatment by Week on the blood values, we additionally performed analyses on the separate time points to assess at which time points the differences occurred. For these individual analyses, a Bonferroni correction was applied, to correct for multiple comparisons.

The effects of treatment on brain weight, relative brain weight (% of body weight at euthanasia), average hippocampal weight (mean of the weight of left and right hippocampus), relative hippocampal weight (% of total brain weight) and iron-containing cell count after iron staining of dorsal hippocampal sections were analyzed using a mixed model ANOVA with the fixed effect Treatment. Hippocampal weights and relative hippocampal weights were log_10_ transformed to meet the normality assumption. In all mixed model analyses, a random effect for litter was added, and the correlation of repeated measures within piglets was addressed using an autoregressive heterogeneous (1) structure for the residuals (SAS PROC MIXED).

Correlations between the linear trend component and general mean of RM scores in the acquisition phase and the iron-containing cell count after staining of each hippocampal area (CA3, CA1, DG, sb) were calculated using Pearsons product-moment correlation coefficient.

## Results

### Weights and growth

**Figure 3:**
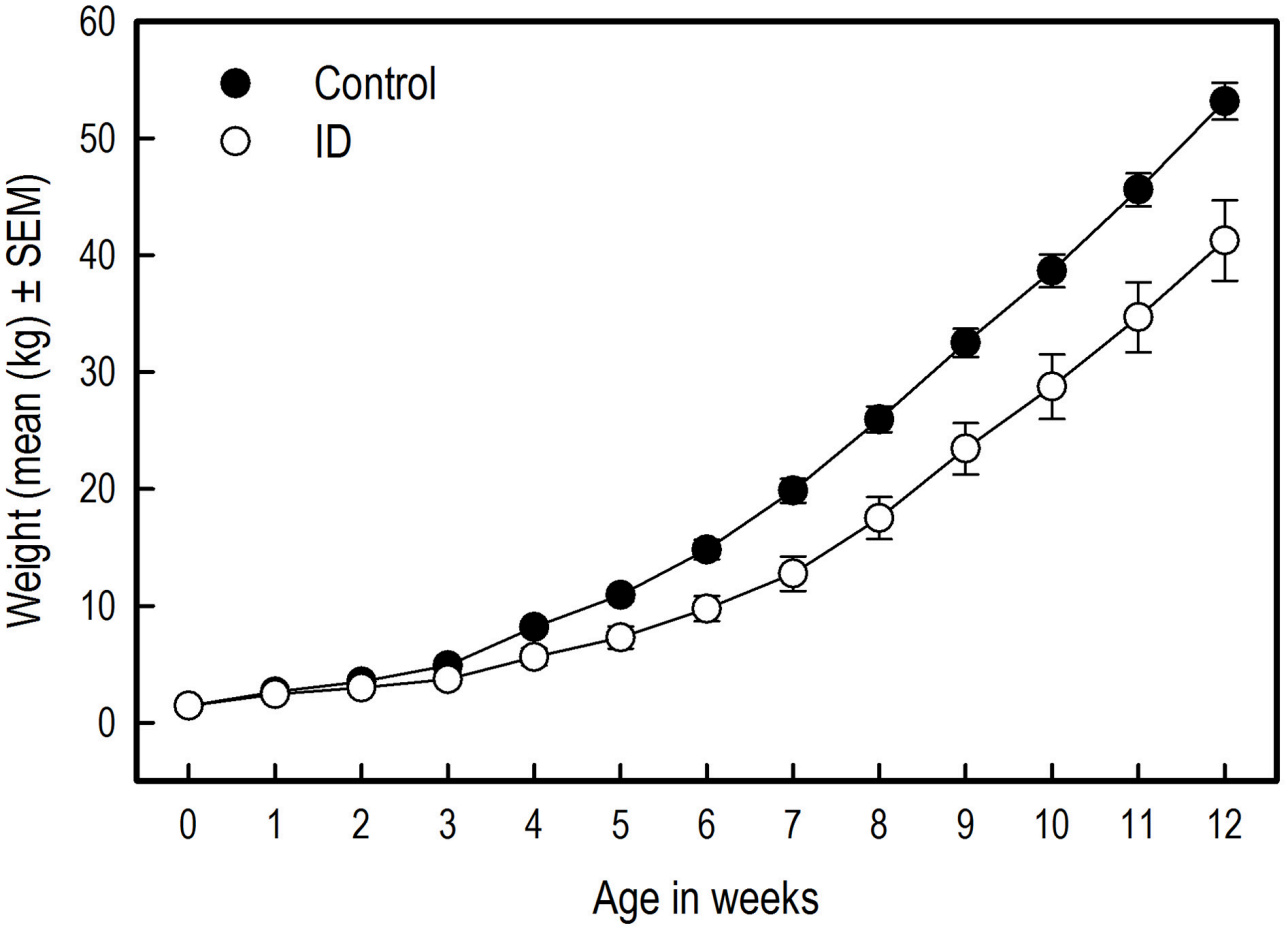
**The body weights of ID (***n*** = 8) and control (***n*** = 10) piglets in kilograms over the course of the experiment**. The difference in body weights between treatment groups was significant from week 3 onward (week 3: *p* = 0.03). Note: The absence of error bars indicates that the SEM is smaller than the plot symbol.

Figure 3 shows the body weights of the animals from birth to 12 weeks of age. The birth weights of the selected piglets did not differ between siblings [*t*_(10)_ = −0.27; *p* = 0.79]. However, over the course of the experiment, the body weights of the control piglets was higher than that of the ID piglets [Treatment: *F*_(1, 203)_ = 22.06; *p* < 0.0001] and control animals grew faster than ID animals [Treatment by Week interaction; *F*_(12, 203)_ = 3.13; *p* = 0.0004]. This difference in body weights between treatment groups was present from week 3 onward [Week 3: *F*_(1, 203)_ = 5.00; *p* = 0.03, and all subsequent weeks with associated *p* < 0.05].

### Holeboard performance

All results of the statistical analyses of the holeboard performance in all phases (habituation, acquisition, transition and reversal) are shown in Supplementary Table 2.

#### Habituation trials

In the habituation phase (six successive trials in which all holes were baited, which preceded formal testing), ID piglets made fewer total visits [Treatment: *F*_(1, 87)_ = 5.70; *p* = 0.02] and found fewer rewards [*F*_(1, 87)_ = 7.72; *p* = 0.01] than control animals. No trial effect or interaction effect was found (Supplementary Table 2A). In the last habituation trial, the number of total visits [Trial: *F*_(5, 87)_ = 2.23; *p* = 0.06, Treatment by Trial interaction: *F*_(1, 87)_ = 0.01; *p* = 0.92] and rewards found [Trial: *F*_(5, 87)_ = 1.44; *p* = 0.22, Treatment by Trial interaction: *F*_(1, 87)_ = 0.01; *p* = 0.91] did not differ between treatment groups (see Supplementary Figure 1). Thus, all piglets searched throughout the entire holeboard for rewards equally effective at the end of the habituation trials.

#### Working memory

##### Acquisition

**Figure 4:**
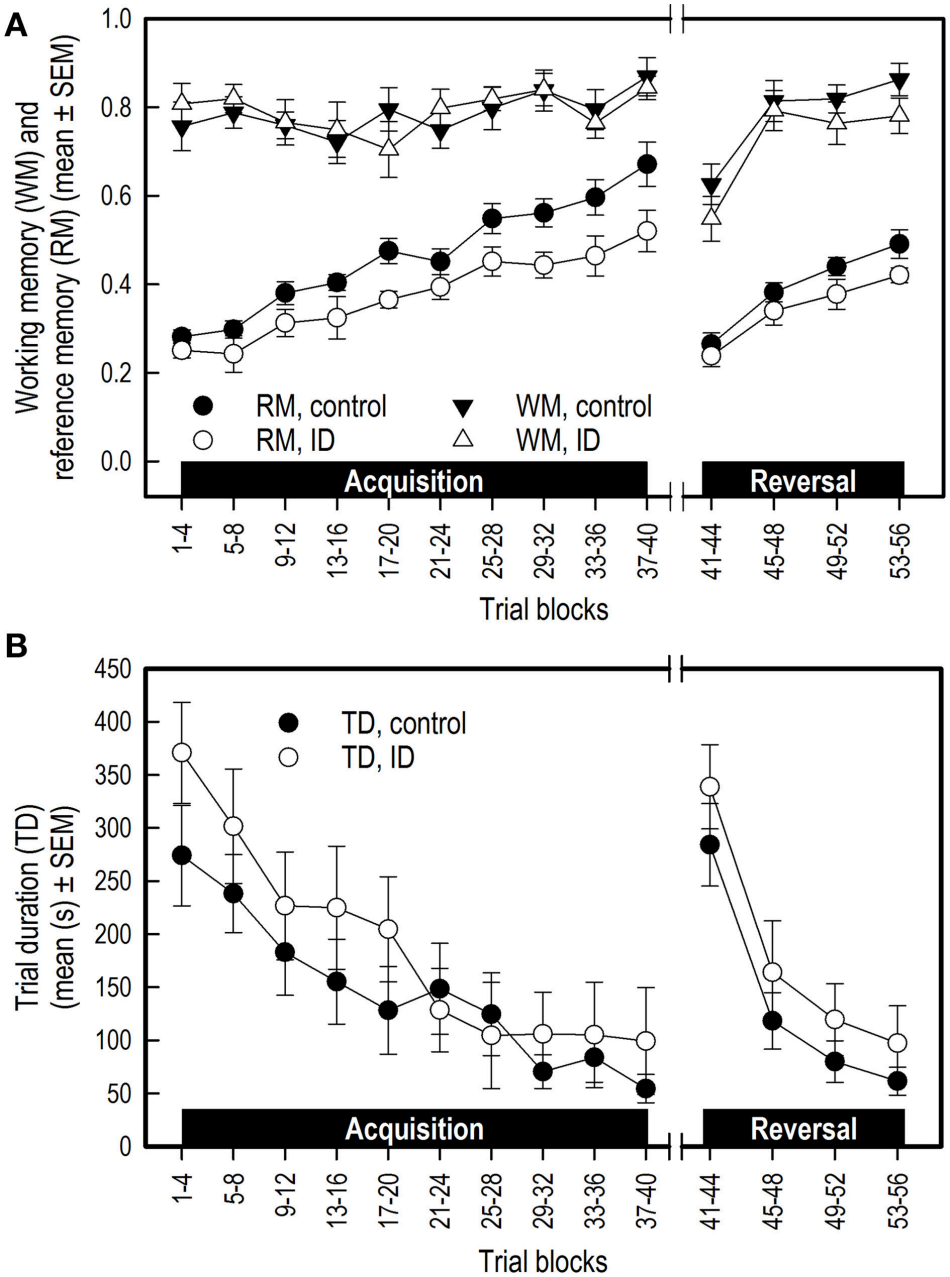
**Performance of ID (***n*** = 8) and control (***n*** = 10) piglets in the spatial cognitive holeboard task during the acquisition phase (trials 1–40) and the reversal phase (trials 41–56)**. **(A)** Working memory (WM) and reference memory (RM) performance. ID animals showed impaired RM performance in the acquisition, transition and reversal phase; see Supplementary Table 2 for results of the statistical analyses. **(B)** Trial duration (TD). Note that TD was analyzed statistically after log_10_ transformation whereas the untransformed means and SEMs are depicted here.

Both groups showed a similar increase in WM performance over the successive acquisition trial blocks [Trial blocks: *F*_(9, 149)_ = 2.17; *p* = 0.03; Figure 4A]. No difference was found in WM in the average performance level between treatment groups, nor an interaction between Treatment and Trial blocks.

##### Transition

When switching from the acquisition to the reversal phase, WM performance dropped similarly in both groups [Trial blocks effect: *F*_(1, 23)_ = 46.25; *p* < 0.0001]. The average WM performance level during transition was unaffected by treatment.

##### Reversal phase

WM performance of both groups increased similarly during the reversal phase [Trial blocks: *F*_(3, 55)_ = 11.41; *p* < 0.0001]. There was a trend that control animals had, on average, a higher WM performance in the reversal phase than the ID animals [*F*_(1, 55)_ = 3.27; *p* = 0.08; Figure 4A]. No interaction between Treatment and Trial blocks was found (Supplementary Table 2B).

#### Reference memory

##### Acquisition

Both groups of piglets showed the same increase in RM performance during acquisition [Trial blocks: *F*_(9, 151)_ = 22.13; *p* < 0.0001]. The control piglets had, on average, higher RM scores than the ID piglets [Treatment: *F*_(1, 151)_ = 17.56; *p* < 0.0001; Figure 4A]. No interaction between Treatment and Trial blocks on RM performance was found.

##### Transition

RM scores dropped in the transition from acquisition to reversal [Trial blocks: *F*_(1, 23)_ = 66.75; *p* < 0.0001]. Although inspection of Figure 4A suggests that the drop in performance of the control group was larger than that of the ID group, this impression was not confirmed statistically [Treatment by Trial blocks interaction: *F*_(1, 23)_ = 2.18; *p* = 0.15]. On average, the RM scores of the control animals during transition were higher than those of the ID piglets [Treatment: *F*_(1, 23)_ = 5.88; *p* = 0.02].

##### Reversal

RM scores increased in the reversal phase for all animals [Trial blocks: *F*_(3, 55)_ = 23.98; *p* < 0.0001]. Control animals had higher average RM scores during this phase than ID animals [Treatment: *F*_(1, 55)_ = 5.14 *p* = 0.03]. No interaction between Treatment and Trial blocks was found (Supplementary Table 2B).

#### Durations and latencies

In both groups, the trial duration (TD), the inter-visit interval (IVI) and the latency until the first reward was found (LFR) decreased over time in both the acquisition phase and reversal phase (Trial blocks effect, Supplementary Table 2B), whereas the latency to the first visit (LFV) only declined in the reversal phase. All duration and latency measures increased in the transition from acquisition to reversal, except for LFV. Treatment had no effect on TD (Figure 4B), IVI, LFV, or LFR in any phase, nor were any interactions found between Treatment and Trial blocks for these measures (Supplementary Table 2B).

#### Number of hole visits

The total number of visits (TV), the number of unrewarded visits (URV) and the number of rewarded visits (RV) did not differ between the groups in any phase (Supplementary Table 2B). For all animals, TV and URV decreased during the acquisition and reversal phase, and increased in the transition from acquisition to reversal. RV only increased in the acquisition phase (Trial blocks, Supplementary Table 2B). No interaction effects were found for these measures.

#### Number of visits before finding the 1st, 2nd, 3rd, and 4th reward

The ID group made more visits in the acquisition phase before finding the first [Treatment: *F*_(1, 149)_ = 8.33; *p* = 0.004] and second [Treatment: *F*_(1, 148)_ = 5.35; *p* = 0.02] reward than the control group did. Additionally, the ID pigs needed more visits to find the fourth reward in the transition to a different set of rewarded holes [Treatment: *F*_(1, 19)_ = 12.75; *p* = 0.002] and during the reversal phase [Treatment: *F*_(1, 51)_ = 15.15; *p* = 0.0003]. The increase in number of visits before finding the fourth reward from the last acquisition to the first reversal trial block was larger in ID piglets than in control animals [Treatment by Trial blocks interaction: *F*_(1, 19)_ = 4.41; *p* = 0.049].

### Blood values

For all three blood parameters hematocrit (Hct) [Treatment by Week interaction: *F*_(4, 58)_ = 14.11; *p* < 0.0001] hemoglobin (Hb) [*F*_(4, 58)_ = 35.90; *p* < 0.0001] and serum iron [*F*_(3, 55)_ = 16.54; *p* < 0.0001], values showed a different curve for the two treatment groups (Supplementary Table 3). In order to investigate at which time points there were treatment effects on these measures, we additionally looked at the effects of treatment per sampling time point (Supplementary Table 4). Because a Bonferroni correction was thus applied, differences with an associated *p* < 0.01 were considered significant in these analyses.

**Figure 5:**
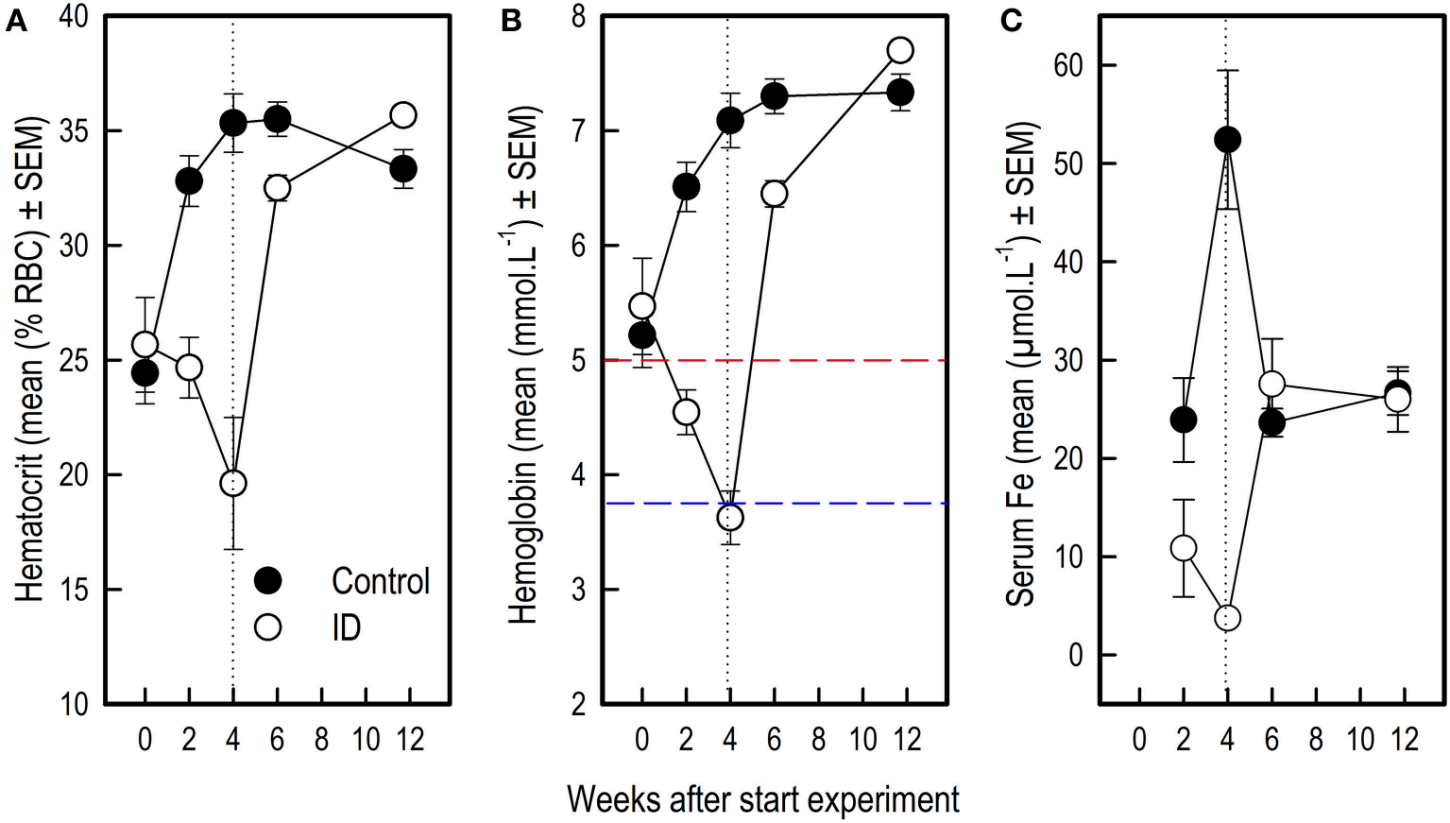
**Blood values in ID and control animals from 0 to 12 weeks after the start of the experiment**. Dietary ID treatment lasted for 4 weeks (dotted black line). **(A)** Hematocrit values (% red blood cells); **(B)** Hemoglobin values. The dashed red line indicates a hemoglobin value of 5, below which piglets are considered anemic, the dashed blue line indicates a value of 3.75, below which piglets are considered severely anemic (Ishaya, 2012); **(C)** Serum iron values. See Supplementary Tables 3, 4 for results of the statistical analyses. Note that the number of observations varied per measure and sampling time point, as reported in Supplementary Table 1.

Before the start of the experimental diet, blood hematocrit [*F*_(1, 58)_ = 0.30; *p* = 0.59] and hemoglobin [*F*_(1, 58)_ = 0.39; *p* = 0.54] values did not differ between siblings. After 2 weeks of dietary treatment, Hct [*F*_(1, 58)_ = 23.24; *p* < 0.0001] and Hb values [*F*_(1, 58)_ = 44.90; *p* < 0.0001] but not serum iron [*F*_(1, 55)_ = 3.42; *p* = 0.07] were lower in the ID group than in the control group. After 4 weeks and thus at the end of treatment, Hct [*F*_(1, 58)_ = 30.79; *p* < 0.0001], Hb [*F*_(1, 58)_ = 134.28; *p* < 0.0001] and serum iron [*F*_(1, 55)_ = 48.29; *p* < 0.0001] were all lower in ID animals than in control animals (Figure 5). Six weeks after the start of treatment, which was 2 weeks after the transition to regular feed, Hct [*F*_(1, 58)_ = 8.82; *p* = 0.004] and Hb [*F*_(1, 58)_ = 17.87; *p* < 0.0001] values were still higher in control animals than in ID animals. Serum iron did not differ between treatment groups at this stage [*F*_(1, 55)_ = 0.87; *p* = 0.35]. At 12 weeks of age, thus 7.5 weeks after treatment and transition to regular feed, ID animals tended to show higher Hct values than control animals [*F*_(1, 58)_ = 5.79; *p* = 0.02], but no differences in Hb [*F*_(1, 58)_ = 3.86; *p* = 0.05] or serum iron [*F*_(1, 55)_ = 0.14; *p* = 0.70] were found between treatment groups (Figure 5; Supplementary Table 4).

### Brain weights

Treatment did not affect the absolute brain weight at 12 weeks of age [*F*_(1, 6)_ = 1.58; *p* = 0.26]. The ID animals had, however, higher brain weights relative to their total body weights (0.20% ± 0.02) than the control animals (0.16% ± 0.005) [*F*_(1, 6)_ = 11.88; *p* = 0.014]. Treatment had no effect on absolute hippocampus weight [*F*_(1, 6)_ = 0.64; *p* = 0.45] nor on the hippocampal weight relative to the total brain weight [*F*_(1, 6)_ = 0.13; *p* = 0.73].

### Brain iron concentrations

**Figure 6:**
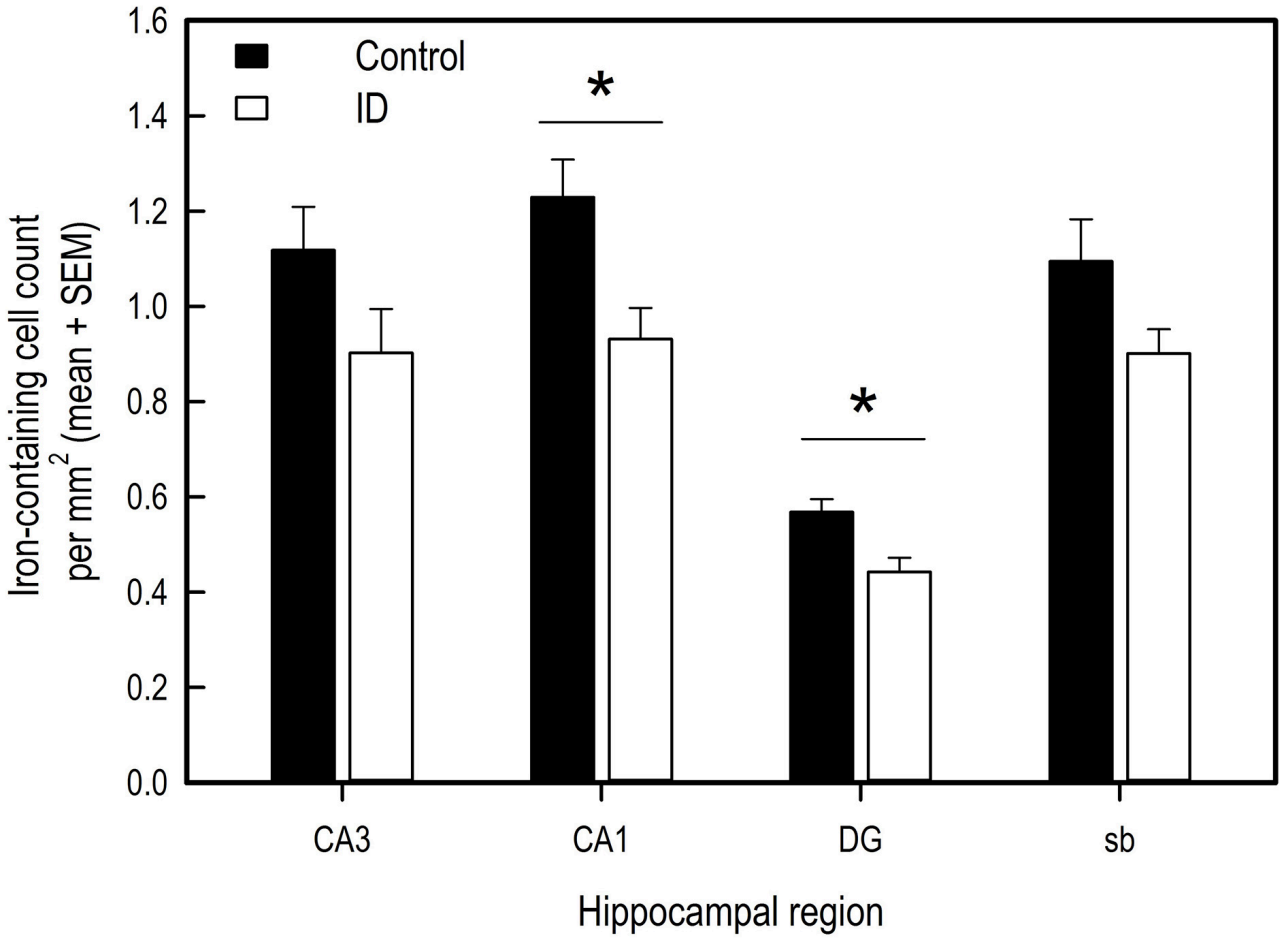
**Number of iron-containing cells in iron stained hippocampal sections of ID (***n*** = 8) and control (***n*** = 9) animals in hippocampal regions CA3, CA1, dentate gyrus (DG) and subiculum (sb) at 12 weeks of age**. ^*^p < 0.05.

**Figure 7:**
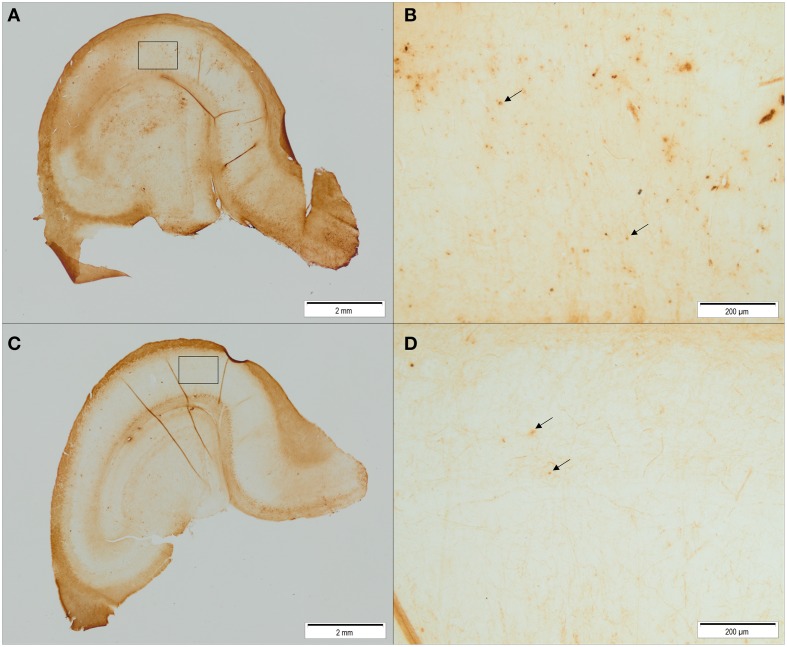
**Representative pictures of iron stained hippocampal sections of control animals (A,B) and ID animals (C,D) at 12 weeks of age**. Sections of ID animals contained significantly fewer iron-containing cells in CA1 (*p* = 0.02) and dentate gyrus (*p* = 0.002) regions than sections of control animals (**A,C**: scale bar = 2 mm, magnification 1.26x; **B,D**: scale bar = 200 μm, magnification 12.6x).

Iron-containing cell count following iron staining showed a difference in iron-containing cells between treatment groups in two hippocampal regions (Figures 6, 7). In the CA3 region, there was no significant difference between control group and ID group [*F*_(1, 6)_ = 3.47; *p* = 0.11]. In the CA1 region, the sections of ID animals contained fewer iron-containing cells compared to sections of control animals [*F*_(1, 6)_ = 10.67; *p* = 0.02]. Similarly, in the DG there were fewer iron-containing cells in ID piglets' sections compared to control piglets' sections [*F*_(1, 6)_ = 25.18; *p* = 0.002]. There was no significant difference in iron-containing cells in the subiculum between the treatment groups [*F*_(1, 6)_ = 3.37; *p* = 0.12].

### Correlations between brain iron and reference memory performance

**Figure 8:**
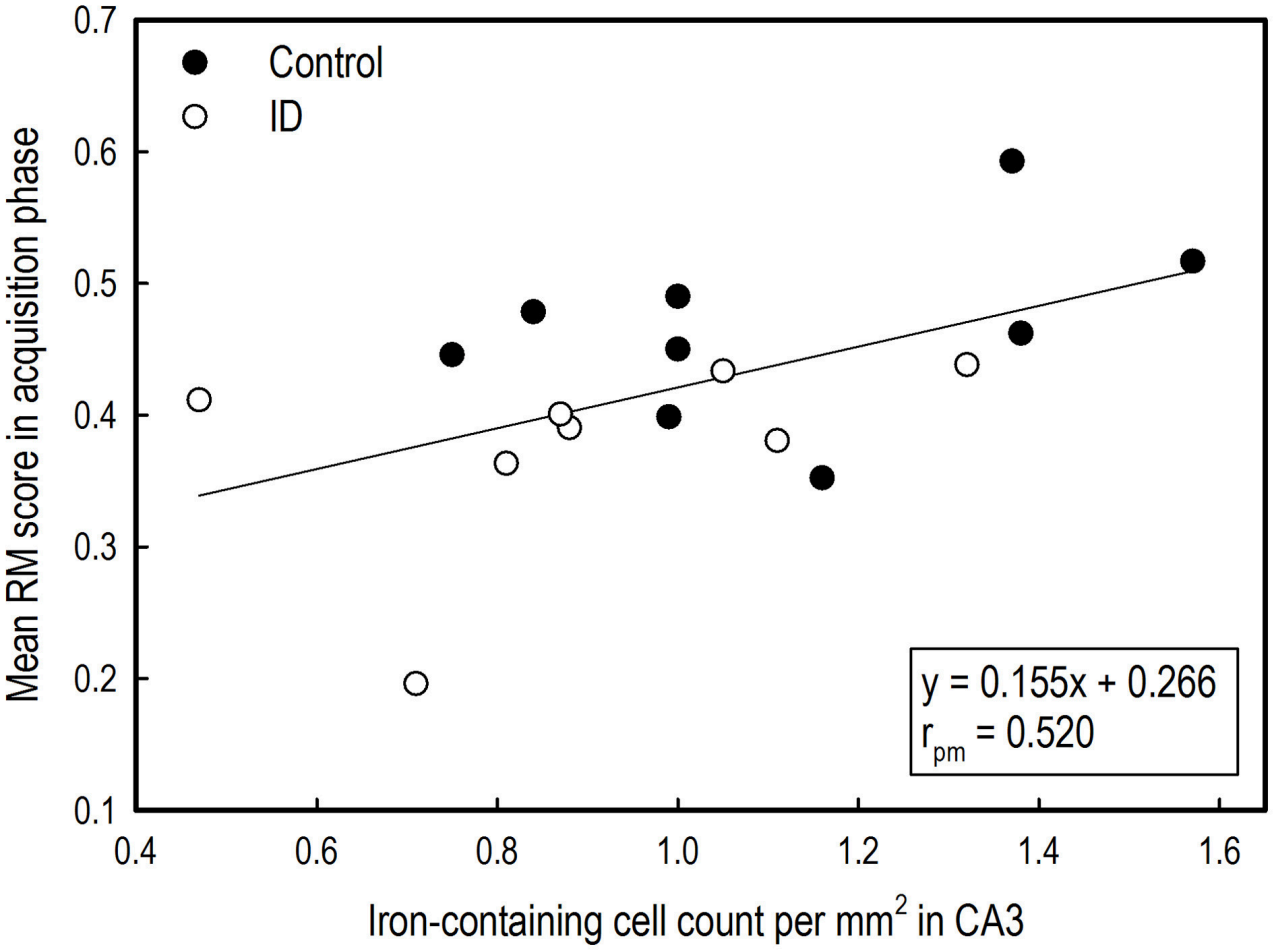
**Correlation between average reference memory scores in the acquisition phase of the holeboard task and iron-containing cell count in hippocampal sections after iron staining of ID (***n*** = 8) and control (***n*** = 9) animals at 12 weeks of age**.

The mean RM scores in the acquisition phase of all animals correlated positively with the number of iron-containing cells in CA3 sections (*r*_pm_ = 0.520; *p* = 0.03; see Supplementary Table 5). This exploratory correlation analysis suggests a moderate relationship between the mean RM performance during acquisition and iron content in the hippocampal CA3 region (Figure 8).

## Discussion

This study investigated the effects of severe early-life iron deficiency on development, cognitive performance, blood parameters and brain histology in piglets later in life. Our results show that severe pre-weaning iron deficiency in piglets causes impairments in physical and mental development, and has long-lasting effects on brain histology.

### Iron deficiency impairs growth

At the start of the experiment, siblings did not differ in birth weight. However, over the course of the experiment, the body weights of the control animals were higher than those of the ID animals and the control animals had a higher growth rate. The weight difference became apparent starting from the third week of age and lasted until the end of the experiment, although iron deficiency treatment had ended at ~5 weeks of age. As we chose to group-house our piglets, we could not measure individual food intake, which ideally should be taken into account in nutritional studies that include growth performance as a read-oud parameter (e.g., Fiset et al., 2015). However, iron deficiency is well-known to impair growth and cause reduced appetite in both rats (Beard et al., 1995) and piglets (Ishaya, 2012). A study investigating the effects of iron deficiency on T-maze performance in piglets found no difference in body weights between ID and control animals (Rytych et al., 2012). However, their study lasted until the age of 28 days, whereas we measured body weights until 12 weeks of age and did not find a difference in weights until 3 weeks of age.

In an iron deficiency study in rats, it was found that impaired growth due to iron deficiency was caused by negative effects on the cell division in growing organs (Canale and Lanzkowsky, 1970). In human infants, iron deficiency causes poor growth and reduced weight gain (Ryan, 1997). Growth is an important indicator of health and nutritional status of children, as disturbances in health and nutrition invariably affect child growth (de Onis et al., 2006). Our data support the notion that iron is an essential micronutrient for neonatal growth and development.

### Impaired memory performance due to iron deficiency treatment

Our study confirms earlier findings that (young) piglets are able to acquire the holeboard task (Arts et al., 2009; Gieling et al., 2012; Elizabeth Bolhuis et al., 2013; Haagensen et al., 2013; Antonides et al., 2015). All piglets acquired the task: their memory scores improved over time and durations and latencies declined over the course of the experiment. During the six habituation trials preceding testing in which all holes were baited, ID piglets made fewer total visits and found fewer rewards than control piglets, i.e., showed less exploration. However, this effect had disappeared by the end of the habituation trials.

This reduced exploration or motivation may be due to the main behavioral symptoms of iron deficiency, such as lethargy, lack of concentration and attentional problems (Lozoff and Brittenham, 1986; Pollitt, 1993). Although we did not record this behavior, ID piglets already seemed to need more time to learn to search for food rewards than control piglets during habituation to the testing environment, i.e., preceding the habituation trials. In an iron deficiency study in rats, it was found that ID rats showed decreased locomotion and a slower rate of habituation (Beard et al., 2002). In future behavioral studies looking at the effects of iron deficiency in pigs, it may therefore be interesting to record behaviors such as time needed for habituation prior to testing.

During the acquisition and reversal phase, ID piglets reached lower reference memory (RM) scores than the control group. These results confirm findings from human studies that a major symptom of iron deficiency is a decline in cognitive performance (Yehuda and Youdim, 1989). The reduced exploration or motivation found in the habituation trials as expressed in fewer total visits and fewer rewards found, was not found in the acquisition or reversal trials. As RM is calculated as a ratio, both components of the ratio would be equally affected by a reduction in exploration or motivation, and thus the outcome would not change. Measures that can serve as indices of motivation or speed, such as trial duration, inter-visit interval, and (latency to the first visit), did not differ between the groups. Moreover, RM performance at the start of both the acquisition and reversal phase did not differ between groups, indicating that both groups started out at the same level of performance in both phases. Similarly, the number of rewarded visits did not differ between groups in any phase. This supports the notion that the reduced performance of the ID animals was a cognitive effect, and was not caused by low motivation to perform the task.

The effects of iron deficiency on several types of cognitive performance have been tested in humans, such as selective attention, working memory, executive function tasks and spatial memory (Lozoff and Georgieff, 2006). With the present study we confirm in particular the finding that iron deficiency leads to impairments in RM performance. This corroborates earlier findings in rats, in which declines in RM scores were found in the Morris water maze up to 4 weeks after iron repletion (Yehuda et al., 1986). Comparably, RM performance was affected in radial arm maze performance of rat pups born to ID mothers (Ranade et al., 2013). In a study investigating the effects of iron deficiency on spatial memory in a double T-maze task in pigs, severe ID piglets could not acquire a food motivated double T-maze task, whereas mild ID piglets showed deficits in reversal learning (Rytych et al., 2012). However, the diets in that study were fed for the total duration of the study, thus piglet performance in the study by Rytych et al. does not provide information about long-term effects of early-life iron deficiency. The piglets in the current study were tested ~3 weeks after treatment ended.

The hematocrit and hemoglobin values of ID pigs two weeks after treatment ended indicate that they may not have been fully iron repleted at the start of behavioral testing, which was 1 week later. However, it may be argued that the slow repletion progress indicates that treatment caused at least some physiological changes to occur, such as a reduced iron uptake ability of ID pigs. Our finding that brain iron was still reduced at 12 weeks of age, which was around 7.5 weeks after treatment ended, is also an indicator that ID pigs were not able to fully recover their ID status within the duration of our study. In an MRI study in pigs, it was found that rapid brain growth in piglets lasts until around 12 weeks of age (Conrad et al., 2012). Thus, although the pig brain is not yet fully grown by that age, most of the growth spurt and brain development has occurred by that time.

To assess whether ID treatment affects cognitive development after this period of rapid brain growth, it may be interesting to monitor and assess physical and cognitive development for a longer period of—or at a later—time. For example, an iron deficiency study in guinea-pigs found decreased locomotor activity at 42 days as compared to 24 days of age (Fiset et al., 2015), suggesting that some detrimental effects of iron deficiency may not show until later in life. Due to a lack of space to house the rapidly growing pigs in our experimental facilities, we could not conduct our study for a prolonged period of time.

Our findings, in combination with previous findings of iron deficiency studies, suggest that early-life iron deficiency causes developmental deficits in the brain. It is suggested that the detrimental effects of iron deficiency on cognition involves the reduction of brain iron, receptor and transporter densities, and changes in serotonin and dopamine pathways (McCann and Ames, 2007; Youdim et al., 2010).

Additional to and in line with the negative effects of iron deficiency on memory scores, ID piglets made more visits before finding the first and second reward in the acquisition phase, and more visits before finding the fourth reward in the transition and reversal phase. When making more errors during a task, memory load is increased, as more information has to be stored for a longer period of time then when fewer errors are made. This increase in errors can therefore be an indication that memory load was increased for ID piglets, or that the ID piglets' executive attention to complete the task was reduced (Gieling, 2013). This is interesting with regard to iron deficiency, as studies in humans have shown that children that suffered early-life iron deficiency display attentional problems (Lozoff and Georgieff, 2006).

WM and TD scores did not differ between the treatment groups in any phase, although ID animals tended to show poorer WM performance in the reversal phase than ID animals. Our results thus support the notion that WM and RM calculated in the manner of this study are independent measures that measure different forms of memory (van der Staay et al., 2012). Visual inspection of the graphical representations of WM and TD results (Figure 4) suggests that the control group had higher WM scores than the ID group in the reversal phase and lower TD over the entire course of the experiment. These impressions, however, were not statistically confirmed.

### Blood values: Anemia in ID animals, iron overload in control animals?

During treatment, all blood iron values decreased in ID animals and increased in control animals. At 12 weeks of age, 7.5 weeks after dietary treatment and transition to regular feed, blood iron values of both treatment groups recovered to similar values. These results show that piglets have some iron stores at birth, which deplete within several weeks after birth if not given iron treatment, either dietary or by means of an iron injection.

In a similar study investigating the effects of early-life iron deficiency on blood parameters in piglets, three groups of piglets were formed: a control group, a mild ID and a severe ID group (Rytych et al., 2012). As in our study, control animals received a 200 mg iron dextran injection. Piglets were separated from the sow 48 h after farrowing, housed individually and fed the assigned milk formula diet. The control group was fed a 100 mg iron/kg diet and the severe ID group 10 mg iron/kg diet, which are similar values as in the current study. Hb values of their ID animals dropped to lower levels as our ID animals after 4 weeks of treatment (2.7 mmol/L compared to 3.7 mmol/L). Hematocrit values followed a comparable decline in their (severe) ID animals and increase in control animals as in the current study.

#### Hematocrit

In a study investigating different administration methods and injection sites of iron on hematocrit (Hct) values in piglets, Hct values varied from 29% at 4 days of age (before iron injection) to 40% at 28 days of age (Koch and Hines, 1969). Different methods and injection sites did not significantly affect Hct values. Buzzard et al. (2013) found average Hct values of 29% in healthy piglets between 3 and 4 weeks of age. Although the values found in the control animals in our study are slightly higher at these ages, their Hct values do fall within the range that Koch and Hines found during and after iron treatment, and the Hct values of our ID animals dropped far below this range to 19% at the end of treatment. After transition to regular feed, Hct values of ID animals recovered to values similar to those of control animals.

#### Hemoglobin

The most common parameter to indicate ID anemia is hemoglobin (Hb; McLean et al., 2009). Hb levels of 6.25 mmol/L or above are assumed normal in piglets, with pigs considered anemic below value of 5 mmol/L. Growth is affected below 4.375 mmol/L and a value below 3.75 mmol/L indicates severe anemia [as converted from g/dL values in (Ishaya, 2012)]. Our results thus imply that all piglets were nearly anemic before treatment, Hb values of control animals rose to normal levels due to iron treatment, and ID animals became severely anemic (3.63 mmol/L ± 0.23) at the end of treatment. Indeed, as Ishaya (2012) argues happens below a Hb value of 4.375, growth of ID animals was impaired, even after treatment had ended (see Section Iron Deficiency Impairs Growth). After transition to regular feed, ID animals' Hb values recovered to normal values, similar to those of control animals.

#### Serum iron

In our control animals, we found surprisingly high values of serum iron at the end of treatment (52.41 μmol/L ± 7.06). High levels of blood serum iron may occur due to iron overload (Cornelius and Kaneko, 1963), which may have been the case in our control animals, as they received both an iron injection and an iron-sufficient diet during treatment. Serum iron of ID animals dropped severely during treatment (3.74 μmol/L ± 0.21), showing that iron reserves were nearly depleted by the end of treatment. Large variation in piglet serum iron values have been reported (Bernát, 1983). In a study investigating factors that influence serum iron in pigs, control animals showed values within the range of 18–35 μmol/L from 0 to 15 weeks of age (Braham et al., 1967), which is in line with the values found in all animals in our study 2 weeks after treatment had ended.

Our results show that piglets are born with low iron stores at birth, which deplete quickly if not given iron treatment, and that an iron administration in common practice may cause iron overload. An excess of iron can be toxic and cause oxidative damage (Lipiñski et al., 2010). In their study, Lipiñski et al. showed that by administrating iron to piglets in two doses at day 3 and 10 instead of in one dose, toxicity can be reduced and iron uptake is improved. Moreover, Yu et al. (2002) argue that a 200 mg iron dextran injection does not contribute to overall performance of piglets at 15 days of age when creep feed is provided from day 7 onward, and consequently may not be a necessity.

### Brain sparing in iron deficient animals

The absolute weights of the brains at 12 weeks of age did not differ between the groups. However, as the ID animals were lighter than the control animals, the relative brain weight compared to total body weight was higher in the ID group than in the control group. This suggests that brain sparing has occurred in the ID animals, meaning that proportionally more energy or nutrients were used for brain development compared to the rest of the body due to their restricted growth (Dobbing, 1971; Hall, 2011; considering the lower brain iron content in ID as opposed to control animals, this does not include the distribution of iron, see Section Early-life Iron Deficiency Reduces Hippocampal Iron Content).

For the weights of hippocampi, no differences were found between the groups. A study looking at a rat iron deficiency model found reduced volumes of the hippocampus in ID rats (Ranade et al., 2013). It is possible that we did not find these effects because we used weight instead of volume.

### Early-life iron deficiency reduces hippocampal iron content

Brain histology after scheduled necropsy at 12 weeks of age showed that in hippocampal regions CA1 and the DG, fewer iron-containing cells were present in ID animals than in control animals. During early development, iron is prioritized to red blood cells at the expense of the brain and other organs when iron supply does not meet the demand (Lozoff and Georgieff, 2006). This may explain why regional brain iron contents, but not blood iron values, were lower in ID pigs than in control animals at 12 weeks of age.

In an iron deficiency study in rat pups, in which a repletion group received an iron-adequate diet after 2 weeks of dietary ID treatment, brain iron and ferritin were normalized after 14 days, whereas brain transferrin was higher than in control animals (Erikson et al., 1997). In their ID group, which received the ID diet for the entire duration of the study, regional brain iron was decreased in cortex and hippocampus. Transferrin concentrations increased drastically in the hippocampi of these ID animals. Transferrin is involved in iron transport and distribution in all tissues, and elevated levels are associated with iron deficiency anemia (Macedo and de Sousa, 2008). In another iron deficiency study with rats that looked at the effects of timing and duration of iron deficiency on brain iron metabolism, 2 weeks of iron repletion after an ID diet from postnatal day 10 to 21 was sufficient to restore (both overall and regional) brain iron concentration (Piñero et al., 2000). It is in comparison to these studies thus surprising that in our piglets we find iron levels to still be lower in ID animals compared to control animals after 7.5 weeks of iron repletion; even when blood iron values had restored to normal. It may be that as the dietary treatment in our study was longer than in the rat studies repletion groups (3 weeks vs. 2 weeks or 11 days, respectively), and at a relatively later developmental stage as rat brains show a different degree of maturation (Romijn et al., 1991), the induced iron deficiency in our ID piglets had stronger negative effects on brain iron concentrations.

The CA1 region plays an important role in short- and intermediate-term memory and self-location (Kesner et al., 2004; Barry and Burgess, 2014). The DG has been suggested to play an important role in learning and memory by processing and representing spatial information (Kesner et al., 2004). Specific destruction of DG cells in rats caused a decrease in performance in a previously acquired radial arm maze task (Walsh et al., 1986; Tilson et al., 1988). In an iron deficiency study that investigated the effects of maternal iron deficiency on rat pup performance, reduced neurogenesis and reduced hippocampal pyramidal and granule cells correlated to impaired radial arm maze performance, in which especially RM scores were affected (Ranade et al., 2013). This correlation between detrimental effects on hippocampal histology and a reduction in memory performance due to iron deficiency is in line with our finding that RM scores correlated positively to iron-containing cells in the CA3 region of the hippocampus.

Our findings that early-life iron deficiency causes both a decrease in memory performance later in life and in brain iron in hippocampal regions that play a role in learning and memory, strongly suggest that iron is essential for these structures to develop and function normally. Although these results may in part be attributed to ID pigs not having fully recovered their iron status at the time of testing, our results do suggest that early-life iron deficiency affects early development of pigs at least for an extended period of time after ID treatment has ended, which has not been shown before in pigs. These findings corroborate previous findings of early-life iron deficiency studies in both humans and rodents.

## Conclusion

Our results show that severe pre-weaning iron deficiency in piglets leads to impaired physical and cognitive development later in life. ID animals showed retarded growth and impaired RM performance in the holeboard task weeks after ID treatment had ended. Our results suggest that early-life iron deficiency affects early development of pigs for an extended period of time. During treatment, blood parameters showed that ID animals became severely anemic, yet their blood values recovered to normal values after transition to iron-sufficient feed after weaning. Serum iron values of control animals showed an indication that iron overload occurred, which may be due to the possibly excessive and toxic administration of 200 mg Fe/kg, which is common in current pig husbandry practice. Brain histology at 12 weeks of age (7.5 weeks after transition to iron-sufficient feed) showed that hippocampal regions CA1 and DG of ID animals had fewer iron-containing cells than those of control animals. The number of iron-containing cells in the CA3 region correlated positively to RM performance in the acquisition phase of all animals. Our findings strongly suggest that iron is essential for brain structures involved in memory and learning to develop and function normally. We conclude that piglets can be used as a model animal to investigate the long-term effects of early-life diets and dietary-induced deficits, and that the holeboard is a sensitive enough task to detect these effects. This task can be used in future research to unravel the pathway through which iron deficiency affects cognition and to develop therapies for treating iron deficiency in both the pig industry as well as in humans.

## Funding

This study was partially funded by Mead Johnson Nutrition (Evansville IN, USA).

### Conflict of interest statement

Mead Johnson participated in designing the study, prepared and coded the synthetic milk diets, but had no influence on the performance of the study, the analyses performed and the interpretation of results. The authors declare that the research was conducted in the absence of any commercial or financial relationships that could be construed as a potential conflict of interest.

## Supplementary material

Supplementary Table 1 **Number of observations per blood collection for blood value analysis**.

Supplementary Table 2 **Performance of ID and control animals in the spatial cognitive holeboard task during habituation (hab) (A), and the acquisition (Acq), transition (Trans), and reversal (Rev) phases (B)**. ^*^For further information about the operational definitions of these variables, see Gieling et al. (2014).

Supplementary Table 3 **Treatment effects on hematocrit, hemoglobin and serum iron of ID and control pigs in a mixed models analysis with Treatment effect, Week effect and their interaction**. See Supplementary Table 4 for effects per sampling time point.

Supplementary Table 4 **Treatment effects on blood values of ID and control pigs per sampling time point during dietary treatment (0–4 weeks) and after transition to regular feed (at 4 weeks of age; dotted line)**. Note that a Bonferroni correction was applied, and that consequently effects of the treatment are considered statistically significant if *p* < 0.01.

Supplementary Table 5 **Correlations between iron-containing cell count in hippocampal areas after iron staining and RM scores during the acquisition phase of the holeboard task**.

Supplementary Figure 1 **The total number of visits (TV) and number of rewards found (REW) in ID (***n*** = 8) and control (***n*** = 10) animals in the six separate habituation trials in the holeboard task, in which all 16 holes were baited with a reward**.
