# Supplementary material for: Pre-weaning dietary iron deficiency impairs spatial learning and memory in the cognitive holeboard task in piglets
Source: Front Behav Neurosci. 2015 Oct 30;9:291. doi: 10.3389/fnbeh.2015.00291 (PMC4626557; doi:10.3389/fnbeh.2015.00291)
Supplement: Supplementary Table 1 — Number of observations per blood collection for blood value analysis. [file Table1.DOCX]

**Supplementary Table 1.** Number of observations per blood collection for blood value analysis.

| **Number of samples per time point of blood collection** | | | | | | |
| --- | --- | --- | --- | --- | --- | --- |
|  |  | | **Age in weeks** | | | |
| **Measure** | **Treatment** | **0** | **2** | **4** | **6** | **12** |
| **Hematocrit (Hct)** | ID | 6 | 8 | 8 | 6 | 6 |
|  | control | 7 | 10 | 9 | 10 | 6 |
| **Hemoglobin (Hb)** | ID | 6 | 8 | 9 | 6 | 6 |
|  | control | 7 | 10 | 9 | 10 | 6 |
| **Serum iron** | ID | ND | 8 | 9 | 8 | 8 |
|  | control | ND | 10 | 10 | 10 | 9 |
