# Supplementary material for: Pre-weaning dietary iron deficiency impairs spatial learning and memory in the cognitive holeboard task in piglets
Source: Front Behav Neurosci. 2015 Oct 30;9:291. doi: 10.3389/fnbeh.2015.00291 (PMC4626557; doi:10.3389/fnbeh.2015.00291)
Supplement: Supplementary Table 2 — Performance of ID and control animals in the spatial cognitive holeboard task during habituation (hab) (A), and the acquisition (Acq), transition (Trans), and reversal (Rev) phases (B). *For further information about the operational definitions of these variables, see Gieling et al. (2014). [file Table2.DOCX]

**Supplementary Table 2.** **Performance of ID and control animals in the spatial cognitive holeboard task** during habituation (hab) (A), and the acquisition (Acq), transition (Trans), and reversal (Rev) phases (B).

| **A) Holeboard habituation (Hab)** | | | | | | | | | | |
| --- | --- | --- | --- | --- | --- | --- | --- | --- | --- | --- |
|  |  | **Treatment** | | | **Trials** | | | **Treatment x Trials** | | |
| **Measure** | **Phase** | **F** | **df** | **P≤** | **F** | **Df** | **P≤** | **F** | **df** | **P≤** |
| **Total number of visits (TV)** | Hab | 5.70 | 1,87 | **0.0191** | 2.23 | 5,87 | 0.0579 | 0.45 | 5,87 | 0.8154 |
| **Number of rewards found (REW)** | Hab | 7.72 | 1,87 | **0.0067** | 1.44 | 5,87 | 0.2192 | 0.44 | 5,87 | 0.8181 |
| ***B) Holeboard acquisition (Acq), transition (Trans), reversal (Rev)*** | | | | | | | | | | |
|  |  | **Treatment** | | | **Trial blocks** | | | **Treatment x Trial blocks** | | |
| **Measure** | **Phase** | **F** | **df** | **P≤** | **F** | **Df** | **P≤** | **F** | **df** | **P≤** |
| **Working memory (WM)** | Acq | 0.00 | 1,149 | 0.9992 | 2.17 | 9,149 | **0.0273** | 0.66 | 9,149 | 0.7460 |
|  | Trans | 1.22 | 1,23 | 0.2799 | 46.25 | 1,23 | **<0.0001** | 0.44 | 1,23 | 0.5119 |
|  | Rev | 3.27 | 1,55 | 0.0761 | 11.41 | 3,55 | **<0.0001** | 0.33 | 3,55 | 0.8018 |
| **Reference memory (RM)** | Acq | 17.56 | 1,151 | **<0.0001** | 22.13 | 9,151 | **<0.0001** | 1.15 | 9,151 | 0.3285 |
|  | Trans | 5.88 | 1,23 | **0.0235** | 66.75 | 1,23 | **<0.0001** | 2.18 | 1,23 | 0.1530 |
|  | Rev | 5.14 | 1,55 | **0.0273** | 23.98 | 3,55 | **<0.0001** | 0.30 | 3,55 | 0.8264 |
| **Trial duration (TD)** | Acq | 0.97 | 1,151 | 0.3258 | 9.66 | 9,151 | **<0.0001** | 1.32 | 9,151 | 0.2290 |
|  | Trans | 1.63 | 1,23 | 0.2144 | 94.09 | 1,23 | **<0.0001** | 0.13 | 1,23 | 0.7199 |
|  | Rev | 1.52 | 1,55 | 0.2225 | 42.51 | 3,55 | **<0.0001** | 0.03 | 3,55 | 0.9915 |
| **Inter-visit-interval (IVI)** | Acq | 0.39 | 1,151 | 0.5320 | 2.19 | 9,151 | **0.0257** | 1.25 | 9,151 | 0.2716 |
|  | Trans | 0.74 | 1,23 | 0.3994 | 24.55 | 1,23 | **<0.0001** | 0.09 | 1,23 | 0.7613 |
|  | Rev | 0.58 | 1,55 | 0.4484 | 18.53 | 3,55 | **<0.0001** | 0.46 | 3,55 | 0.7088 |
| **Latency first visit (LFV)** | Acq | 0.60 | 1,151 | 0.4399 | 0.79 | 9,151 | 0.6279 | 0.30 | 9,151 | 0.9744 |
|  | Trans | 0.15 | 1,23 | 0.6976 | 0.30 | 1,23 | 0.5866 | 0.04 | 1,23 | 0.8476 |
|  | Rev | 0.53 | 1,55 | 0.4697 | 3.17 | 3,55 | **0.0313** | 0.24 | 3,55 | 0.8711 |
| **Latency first rewarded visit (LFR)** | Acq | 2.70 | 1,151 | 0.1022 | 4.32 | 9,151 | **<0.0001** | 0.52 | 9,151 | 0.8558 |
|  | Trans | 0.67 | 1,23 | 0.4215 | 34.30 | 1,23 | **<0.0001** | 0.20 | 1,23 | 0.6561 |
|  | Rev | 0.73 | 1,55 | 0.3967 | 12.96 | 3,55 | **<0.0001** | 0.11 | 3,55 | 0.9546 |
| **Total number of visits (TV)** | Acq | 0.12 | 1,151 | 0.7297 | 5.33 | 9,151 | **<0.0001** | 1.18 | 9,151 | 0.3124 |
|  | Trans | 1.29 | 1,23 | 0.2679 | 100.92 | 1,23 | **<0.0001** | 0.01 | 1,23 | 0.9057 |
|  | Rev | 1.74 | 1,55 | 0.1931 | 15.12 | 3,55 | **<0.0001** | 1.22 | 3,55 | 0.3109 |
| **Unrewarded visits (URV)** | Acq | 0.71 | 1,151 | 0.3991 | 7.11 | 9,151 | **<0.0001** | 1.16 | 9,151 | 0.3268 |
|  | Trans | 1.27 | 1,23 | 0.2715 | 118.57 | 1,23 | **<0.0001** | 0.00 | 1,23 | 0.9670 |
|  | Rev | 2.48 | 1,55 | 0.1213 | 21.72 | 3,55 | **<0.0001** | 1.16 | 3,55 | 0.3324 |
| **Rewarded visits (RV)** | Acq | 1.33 | 1,151 | 0.2510 | 2.11 | 9,151 | **0.0322** | 0.83 | 9,151 | 0.5867 |
|  | Trans | 0.02 | 1,23 | 0.8826 | 2.63 | 1,23 | 0.1182 | 0.28 | 1,23 | 0.6025 |
|  | Rev | 0.12 | 1,55 | 0.7309 | 0.64 | 3,55 | 0.5948 | 1.02 | 3,55 | 0.3895 |
| **Rewards found (REW)** | Acq | 0.63 | 1,151 | 0.4280 | 1.57 | 9,151 | 0.1296 | 0.96 | 9,151 | 0.4765 |
|  | Trans | 1.01 | 1,23 | 0.3261 | 24.92 | 1,23 | **<0.0001** | 0.11 | 1,23 | 0.7423 |
|  | Rev | 0.70 | 1,55 | 0.4067 | 9.90 | 3,55 | **<0.0001** | 0.32 | 3,55 | 0.8133 |
| **Visits before 1^st^ reward (Vfirst)*** | Acq | 8.33 | 1,149 | **0.0045** | 6.16 | 9,149 | **<0.0001** | 0.41 | 9,149 | 0.9302 |
|  | Trans | 0.47 | 1,23 | 0.5004 | 15.81 | 1,23 | **0.0006** | 0.85 | 1,23 | 0.3664 |
|  | Rev | 0.09 | 1,55 | 0.7706 | 4.62 | 3,55 | **0.0059** | 0.48 | 3,55 | 0.6968 |
| **Visits before 2^nd^ reward (Vsecond)*** | Acq | 5.35 | 1,148 | **0.0221** | 7.79 | 9,148 | **<0.0001** | 0.45 | 9,148 | 0.9061 |
|  | Trans | 1.26 | 1,23 | 0.2725 | 37.13 | 1,23 | **<0.0001** | 2.71 | 1,23 | 0.1132 |
|  | Rev | 1.09 | 1,55 | 0.3014 | 9.52 | 3,55 | **<0.0001** | 1.06 | 3,55 | 0.3740 |
| **Visits before 3^rd^ reward (Vthird)*** | Acq | 2.08 | 1,144 | 0.1511 | 8.11 | 9,144 | **<0.0001** | 0.65 | 9,144 | 0.7532 |
|  | Trans | 0.38 | 1,21 | 0.5439 | 40.46 | 1,21 | **<0.0001** | 0.00 | 1,21 | 0.9713 |
|  | Rev | 0.56 | 1,52 | 0.4595 | 9.26 | 3,52 | **<0.0001** | 0.11 | 3,52 | 0.9548 |
| **Visits before 4^th^ reward (Vfourth)*** | Acq | 1.27 | 1,131 | 0.2612 | 10.07 | 9,131 | **<0.0001** | 1.76 | 9,131 | 0.0816 |
|  | Trans | 12.75 | 1,19 | **0.0020** | 39.14 | 1,19 | **<0.0001** | 4.41 | 1,19 | **0.0492** |
|  | Rev | 15.15 | 1,51 | **0.0003** | 10.31 | 3,51 | **<0.0001** | 1.86 | 3,51 | 0.1486 |

*: For further information about the operational definitions of these variables, see Gieling et al., 2014.
