# Supplementary material for: Pre-weaning dietary iron deficiency impairs spatial learning and memory in the cognitive holeboard task in piglets
Source: Front Behav Neurosci. 2015 Oct 30;9:291. doi: 10.3389/fnbeh.2015.00291 (PMC4626557; doi:10.3389/fnbeh.2015.00291)
Supplement: Supplementary Table 3 — Treatment effects on hematocrit, hemoglobin and serum iron of ID and control pigs in a mixed models analysis with Treatment effect, Week effect and their interaction. See Supplementary Table 4 for effects per sampling time point. [file Table3.DOCX]

**Supplementary Table 3.** **Treatment effects on hematocrit, hemoglobin and serum iron of ID and control pigs** in a mixed models analysis with Treatment effect, Week effect and their interaction. See Supplementary Table 4 for effects per sampling time point.

| ***Treatment effects on blood parameters*** | | | | | |  | |  | |  | |
| --- | --- | --- | --- | --- | --- | --- | --- | --- | --- | --- | --- |
| **Blood parameter** | **Treatment** | | | **Week** | | | **Treatment x Week** | | | | |
|  | **F** | **df** | **P≤** | **F** | **df** | **P≤** | **F** | | **df** | | **P≤** |
| ***Hematocrit*** | 21.75 | 1,58 | **<0.0001** | 19.63 | 4,58 | **<0.0001** | 14.11 | | 4,58 | | **<0.0001** |
| ***Hemoglobin*** | 38.01 | 1,58 | **<0.0001** | 51.96 | 4,58 | **<0.0001** | 35.90 | | 4,58 | | **<0.0001** |
| ***Serum iron*** | 25.91 | 1,55 | **<0.0001** | 2.39 | 3,55 | 0.0790 | 16.54 | | 3,55 | | **<0.0001** |
