# Supplementary material for: Pre-weaning dietary iron deficiency impairs spatial learning and memory in the cognitive holeboard task in piglets
Source: Front Behav Neurosci. 2015 Oct 30;9:291. doi: 10.3389/fnbeh.2015.00291 (PMC4626557; doi:10.3389/fnbeh.2015.00291)
Supplement: Supplementary Table 4 — Treatment effects on blood values of ID and control pigs per sampling time point during dietary treatment (0–4 weeks) and after transition to regular feed (at 4 weeks of age; dotted line). Note that a Bonferroni correction was applied, and that consequently effects of the treatment are considered statistically significant if p < 0.01. [file Table4.DOCX]

**Supplementary Table 4.** **Treatment effects on blood values of ID and control pigs per sampling time point** during dietary treatment (0-4 weeks) and after transition to regular feed (after 4 weeks of treatment; dotted line). Note that a Bonferroni correction was applied, and that consequently effects of the treatment are considered statistically significant if p < 0.01.

| ***Effects of treatment per sampling time point*** | | | | |  |  |  |  |  |
| --- | --- | --- | --- | --- | --- | --- | --- | --- | --- |
| **Treatment** | **Hematocrit** | | | **Hemoglobin** | | | **Serum iron** | | |
| **wk** | **F** | **df** | **P≤** | **F** | **df** | **P≤** | **F** | **df** | **P≤** |
| 0 | 0.30 | 1,58 | 0.5888 | 0.39 | 1,58 | 0.5362 | - | - | - |
| 2 | 23.24 | 1,58 | **<0.0001** | 44.90 | 1,58 | **<0.0001** | 3.42 | 1,55 | 0.0697 |
| 4 | 30.79 | 1,58 | **<0.0001** | 134.28 | 1,58 | **<0.0001** | 48.29 | 1,55 | **<0.0001** |
| 6 | 8.82 | 1,58 | **0.0043** | 17.87 | 1,58 | **<0.0001** | 0.87 | 1,55 | 0.3538 |
| 12 | 5.79 | 1,58 | 0.0193 | 3.86 | 1,58 | 0.0543 | 0.14 | 1,55 | 0.7050 |
