# Supplementary material for: Pre-weaning dietary iron deficiency impairs spatial learning and memory in the cognitive holeboard task in piglets
Source: Front Behav Neurosci. 2015 Oct 30;9:291. doi: 10.3389/fnbeh.2015.00291 (PMC4626557; doi:10.3389/fnbeh.2015.00291)
Supplement: Supplementary Table 5 — Correlations between iron-containing cell count in hippocampal areas after iron staining and RM scores during the acquisition phase of the holeboard task. [file Table5.DOCX]

**Supplementary Table 5.** Correlations between iron-containing cell count in hippocampal areas after iron staining and RM scores during the acquisition phase of the holeboard task.

| **Correlations between iron-containing cell count in hippocampus and RM scores** | | | | | |
| --- | --- | --- | --- | --- | --- |
| **All animals (n=17)** |  | **CA3** | **CA1** | **DG** | **sb** |
| **RM acquisition linear trend** | r_pm_ | 0.390 | 0.120 | 0.246 | 0.214 |
|  | p< | 0.122 | 0.647 | 0.341 | 0.409 |
| **RM acquistion mean** | r_pm_ | 0.520 | 0.160 | 0.193 | 0.239 |
|  | p< | **0.032** | 0.540 | 0.457 | 0.355 |
| **RM reversal linear trend** | r_pm_ | 0.386 | 0.061 | 0.108 | -0.001 |
|  | p< | 0.126 | 0.816 | 0.680 | 0.997 |
| **RM reversal mean** | r_pm_ | 0.258 | 0.069 | 0.075 | 0.147 |
|  | p< | 0.317 | 0.793 | 0.775 | 0.573 |
| **RM difference score** | r_pm_ | 0.464 | 0.162 | 0.118 | 0.244 |
|  | p< | 0.061 | 0.535 | 0.652 | 0.345 |
