# Supplementary figures and images for: Pre-weaning dietary iron deficiency impairs spatial learning and memory in the cognitive holeboard task in piglets
Source: Front Behav Neurosci. 2015 Oct 30;9:291. doi: 10.3389/fnbeh.2015.00291 (PMC4626557; doi:10.3389/fnbeh.2015.00291)

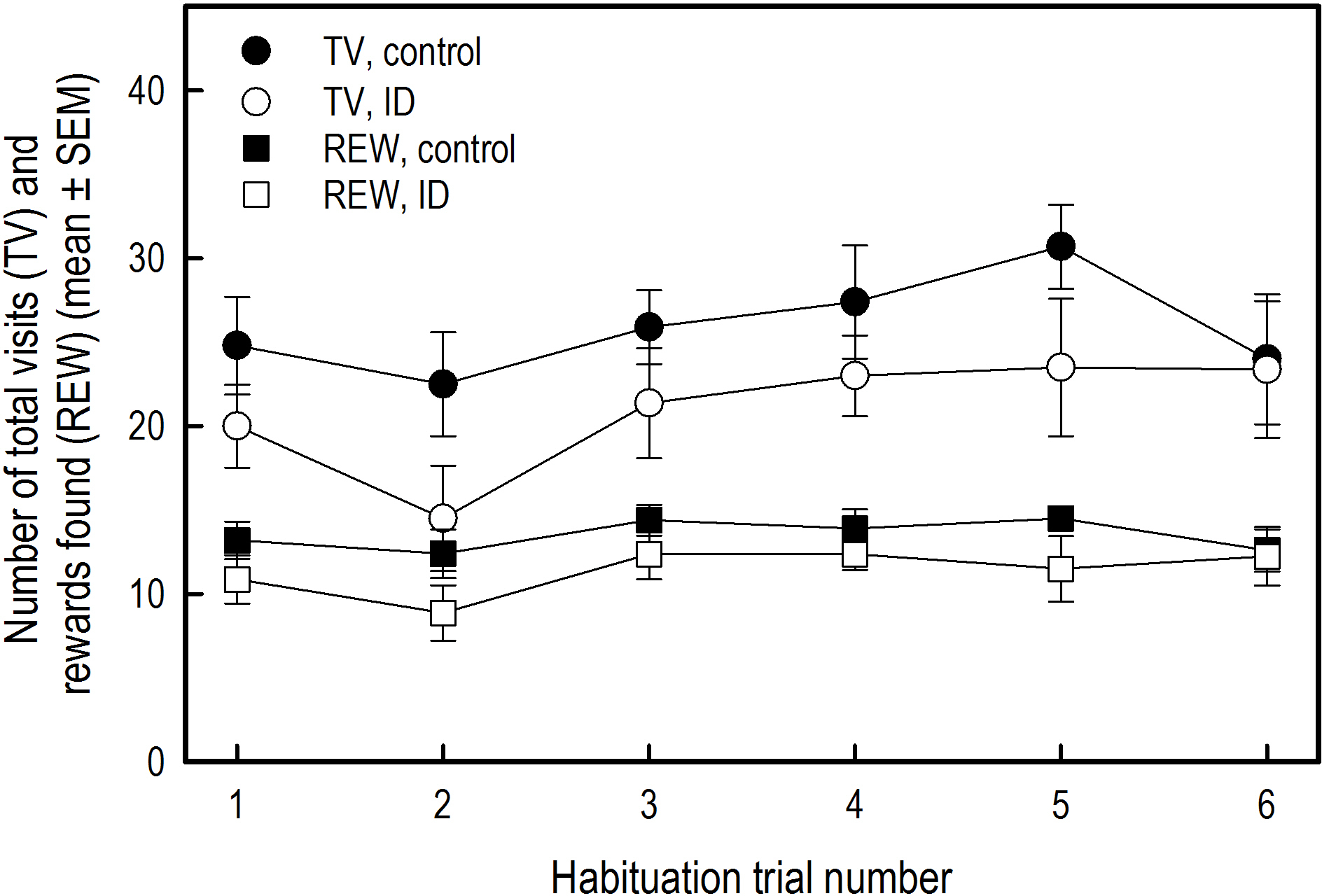

Supplement: Supplementary Figure 1 — The total number of visits (TV) and number of rewards found (REW) in ID (n = 8) and control (n = 10) animals in the six separate habituation trials in the holeboard task, in which all 16 holes were baited with a reward. [file Image1.JPEG]
